# Influencing factors of stroke occurrence and recurrence in hypertensive patients: A prospective follow‐up studies

**DOI:** 10.1002/brb3.2770

**Published:** 2022-09-13

**Authors:** Yuelong Jin, Minmin Jiang, Na Pan, Yan Chen, Weiwei Chang, Lijun Zhu, Zhengmei Fang, Shizao Fei, Zixuan Zhou, Siyun Zhou, Lianping He, Yingshui Yao

**Affiliations:** ^1^ School of Public Health Institute of Chronic Disease Control and Prevention, Wannan Medical College Wuhu 241002 Anhui China; ^2^ The Second People's Hospital Wuhu 241001 Anhui China; ^3^ School of Medicine Taizhou University Jiaojiang 318000 Zhejiang China; ^4^ Anhui College of Traditional Chinese Medicine Wuhu 241003 Anhui China

**Keywords:** hypertension, influence factors, occurrence, recurrence, stroke

## Abstract

**Objective:**

To understand the related risk factors of occurrence and recurrence of hypertension and provide scientific basis for relevant departments to better guide prevention and control work.

**Methods:**

From September 2017 to September 2018, a prospective follow‐up study was performed on patients with hypertension who visited the Second People's Hospital of Wuhu City, Anhui Province. Multivariate Cox regression was used to analyze influencing factors of stroke occurrence and recurrence in follow‐up of hypertensive patients.

**Results:**

A total of 769 hypertensive patients were enrolled in this study. The average age of hypertensive patients was 65.66 ± 11.70 years old and the BMI index was 24.99 ± 4.17. In this study, 769 patients with hypertension were followed up for 1 year, and the incidence of stroke was 14.69%. This study found that higher levels of blood glucose (RR = 2.027, 95% CI: 1.195–3.438), HCY (RR = 5.928,95% CI: 1.438–24.440), aggravated extent of carotid artery stenosis (RR = 2.620, 95% CI: 1.532–4.481), and drinking (RR = 3.867, 95% CI: 2.038–7.339) were risk factors, and maintaining exercise (RR = 0.325, 95% CI: 0.117–0.907) was a protective factor for stroke occurrence; however, aggravated extent of carotid artery stenosis (RR = 3.158, 95% CI: 1.797–5.550) and smoking (RR = 2.271, 95% CI: 1.142–4.517) were risk factors for stroke recurrence for hypertensive patients.

**Conclusions:**

For people with high blood pressure, it is necessary to exercise properly, control body weight, avoid obesity, quit smoking, reduce alcohol consumption, and reasonably control blood pressure, blood sugar, and blood lipid.

## Graphical abstract

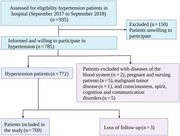

## 1 INTRODUCTION

Stroke is defined as “rapidly developing signs of focal (or global) disturbance of cerebral function lasting >24 h (unless interrupted by surgery or death) with no apparent nonvascular cause” (Hatano, 1976). According to some research results, stroke is the second leading cause of death in the world and is recognized as the major cause of acquired disability for adults in most regions (Lozano et al., 2012; O'Donnell & Yusuf, 2009). Strokes can usually be divided into two subgroups: ischemic stroke and hemorrhagic stroke. Although over the past several decades, age‐standardized stroke mortality and morbidity have continued to decline in many developed countries (Kim et al., 2015), stroke burden has increased over the last 30 years in China, and the age‐standardized prevalence, incidence and mortality rates were 1114.8 per 100,000 people, 246.8 and 114.8 per 100,000 person‐years, respectively (Wang et al., 2017).Stroke not only endangers the life and health of patients and reduces their quality of life, but also brings a huge economic and medical burden to the patients' families and society. Stroke accounts for about 2%–4% of total world health care costs, and industrialized countries have shown that stroke accounts for 4% of direct health care costs (Li et al., 2019).

Reducing the burden of stroke in the population requires identifying modifiable risk factors and demonstrating the effectiveness of risk reduction efforts. There are many risk factors for stroke, including both modifiable (e.g., physical exercise, BMI, diet, smoking, drinking, comorbid conditions) and nonmodifiable risk factors (e.g., age, sex, race) (Boehme et al., 2017). Studies have shown that hypertension is the common most and powerful modifiable risk factor for stroke regardless of geographic region and ethnicity, though it contributes to atherosclerotic disease (Boehme et al., 2017; Iadecola & Gorelick, 2004). Evidence from clinical trials shows that controlling of blood pressure can substantially lower risk of stroke (Chobanian et al., 2003).

Early detection, diagnosis, and treatment are critical to prevent and control stroke. Therefore, regular health examination is an important method for early detection of people at risk of stroke. In the present study, we conducted a 1‐year follow‐up of hypertension patients at the Second People's Hospital of Wuhu City, Anhui Province to reveal the distribution characteristics of risk of stroke among the hypertensive population. The factors related to the occurrence and recurrence risk of stroke were discussed to provide scientific basis and effective data support for better guiding the prevention and treatment of stroke.

## 2 SUBJECTS AND METHODS

### 2.1 Study design and participants

**FIGURE 1:**
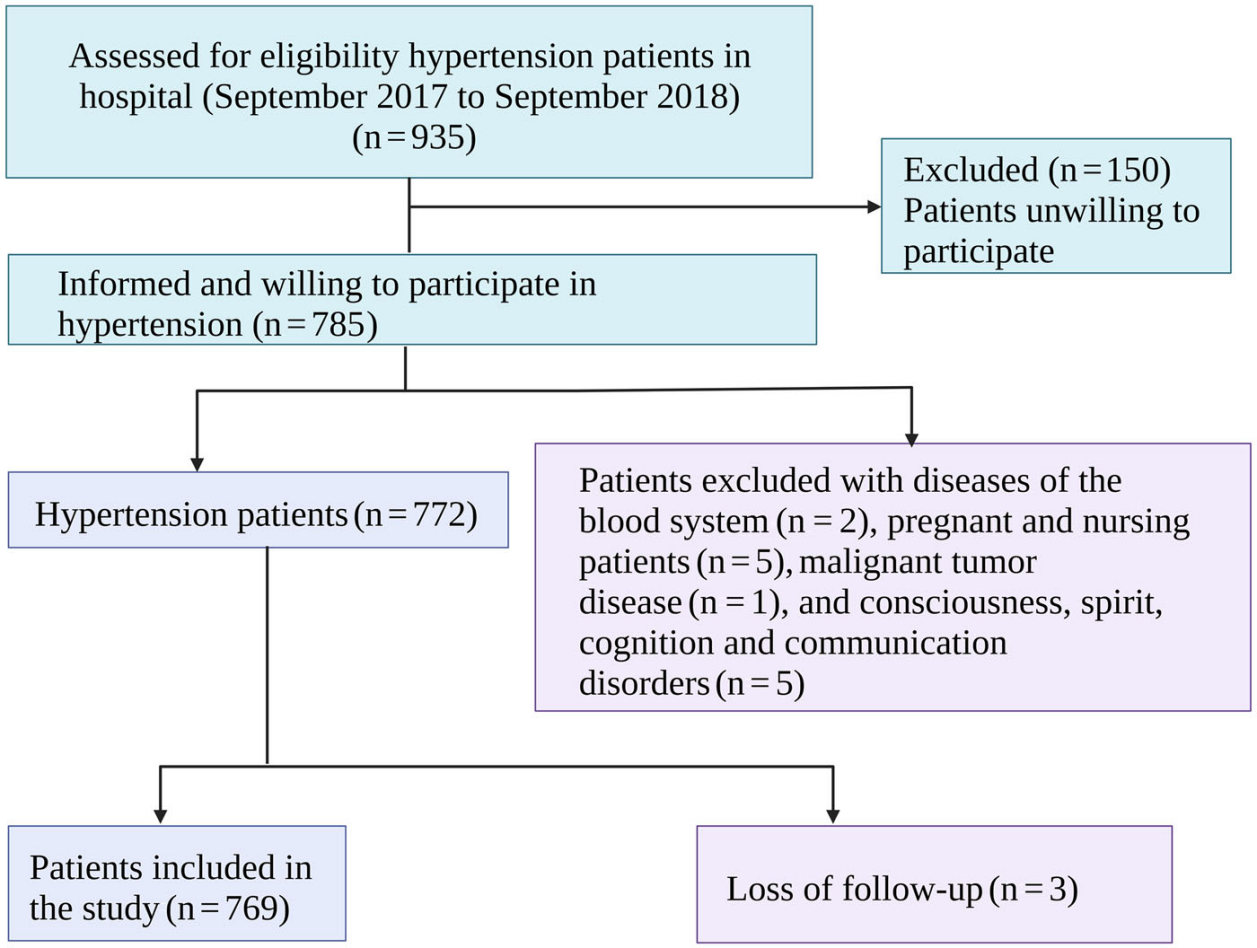
The selection flow chart of the study population

This was a prospective follow‐up study of hospital hypertensive patients. All hypertensive patients admitted to the second people's hospital of Wuhu city, Anhui province from September 2017 to September 2018 were selected as subjects. All the patients were from Wuhu district, Anhui province and recorded as the admission number, age, gender, detailed relevant factors including history of stroke, history of diabetes, history of hyperlipidemia, smoking, drinking, and exercise behavior, etc. The inclusion criteria of the subjects are as follows: (1) the subjects have the complete general information and inpatient information;(2) the subjects volunteered to cooperate with the inspection and study, and signed the informed consent; and (3) hypertensive patients. Exclusion criteria are as follows: (1) patients with diseases of the blood system; (2) patients with severe lesions of organs and tissues; (3) pregnant and nursing patients; (4) patients with malignant tumor disease; (5) patients with poor coordination; and (6) patients with consciousness, spirit, cognition, and communication disorders. The selection flow chart of the study population is shown in Figure 1.

This study was approved by the Ethics Committee of the Second People's Hospital of Wuhu (No.2021027).

### 2.2 Baseline survey

According to the 2017 technical program of screening and intervention project for high‐risk groups of stroke, all hypertensive patients enrolled during the study period were comprehensively evaluated and screened by means of questionnaire survey, physical examination, and laboratory examination.

### 2.3 Questionnaire survey

The self‐designed questionnaire was used, and the face‐to‐face survey was conducted by uniformly trained investigators. The contents of the survey included (1) basic information: gender; age; disease history of stroke, diabetes, and hyperlipidemia; smoking; drinking; physical activity. Physical activity included medium or heavy manual workers and/or exercise with at least moderate intensity for more than half an hour and no <3 times a week. Smoking was defined as smoking ≥1 cigarette per day for 1 year or more, including current smokers and ex‐smokers, smoking. Any of the following case is defined as drinking: ① frequent heavy drinking refers to white wine ≥3 times/week, ≥100 g each time; ② there used to be drinking habits, but now abstains from drinking; ③ have drinking habits, but rarely in large quantities.

### 2.4 Physical examination

Body measurements include height, weight, and blood pressure. Height measurement requires the subjects to take off their shoes and hats, keep their heels tight and upright, and fix them vertically on the wall with a tape measure. The nurses use a triangular plate to read the right angle side against the wall, and the degree is accurate to 0.5 cm. The weight measurement requires subjects to take off shoes and coats, wear only single clothing, and the degree is accurate to 0.5 kg. The body mass index (BMI) was calculated by dividing the weight in kilograms by the squared height in meters (kg/m^2^). Blood pressure was measured using a mercury sphygmomanometer in a separate room in a quiet environment. The subjects need to sit still for 5 min before measurement and take the average value for 3 consecutive measurements. The interval between any two blood pressure measurements should be more than 30 s. Carotid artery stenosis (CAS) was measured by magnetic resonance imaging (MRI). According to the degree of CAS, the patients were classified into mild, moderate, and severe stenosis group.

### 2.5 Laboratory examination

5 ml of fasting venous blood was taken from the subjects in the morning, and the levels of total cholesterol (TC), triglycerides (TG), high‐density lipoprotein (HDL‐C), low‐density lipoprotein (LDL‐C), homocysteine (HCY), and blood glucose were measured.

### 2.6 Research indicators and criteria

Hypertension is defined as any of the following: (1) blood pressure ≥140 and/or 90 mmHg; (2) currently taking antihypertensive drugs; (3) self‐report that he/she has been diagnosed with hypertension by doctors. Diabetes was defined as blood glucose ≥ 7.0/11.0 mmol/L (not eating/other time) or currently under medication control.

Dyslipidemia was defined as one or more abnormal indexes (TC ≥ 6.22 mmol/L, TG ≥ 2.26 mmol/L, HDL‐C < 1.04 mmol/L, LDL‐C ≥ 4.14 mmol/L) or lipid‐lowering drugs being used. According to the guidelines for the prevention and control of overweight and obesity in Chinese adults, 24 kg/m^2^ ≤ BMI ≤ 28 kg/m^2^ is defined as overweight and BMI ≥ 28 kg/m^2^ is defined as obesity. The diagnostic criteria of stroke are that patients have typical clinical symptoms and evidence of head MRI.

### 2.7 Intervention and follow‐up outcomes

A 1‐year intervention was carried out for the hypertensive patients enrolled, including relevant knowledge lectures, distribution of health knowledge brochures related to stroke, implementation of low‐salt, low‐sugar, and low‐fat diet plan, guidance of patients to quit smoking and drinking, reasonable work and rest system and guiding overweight and obese patients for weight control and reasonable exercise, etc.

Starting from the date of admission, the patients were followed up for 6 months and 1 year, with a total of 2 times. Follow‐up was mainly by telephone, supplemented by outpatient follow‐up. Patients who have lost contact for various reasons, such as the mobile phone number change, shutdown, three or more phone calls or refusal to accept follow‐up are regarded as shedding cases. The main contents of the follow‐up included stroke incidence and death. The endpoint observed was stroke or stroke recurrence during follow‐up.

### 2.8 Quality control

Before the investigation, an Expert Symposium was carried out to discuss the specific implementation steps of the project, formulate the investigator manual and stipulate unified standards. All graduate students who participated in the survey received unified professional training. Before the implementation of formal on‐site investigation, we selected a small range of people for pre investigation to find problems in time. After improvement and training, the on‐site investigation was started. The whole process of on‐site investigation shall be supervised by the designated personnel of the leading group. After the questionnaire is collected, the leading group shall check whether there are errors and omissions and timely change and make up for errors and omissions.

### 2.9 Statistical analysis

The SPSS18.0 statistical software program was used for data analysis. Quantitative data and categorical data were expressed by mean with SD and frequency or percentage, respectively. The incidence and recurrence rate of stroke in different group (gender, age, history of stroke, smoking, etc.) were compared by using the chi‐squared (χ^2^) tests and exact probability method of Fisher. The independent variables with statistical significance (*p* value < .1) in univariate analysis (χ^2^ test) were included in multivariate Cox regression to further analyze the risk factors of new onset and recurrence of stroke. *p* Value < .05 was considered significant.

## 3 RESULTS

### 3.1 General clinical characteristics, smoking, drinking, and exercise behavior of hypertensive patients

A total of 769 hypertensive patients were included in this study. Their general clinical characteristics, smoking, drinking, and exercise behaviors were shown in Table 1. The average age of hypertensive patients was 65.66 ± 11.70 years; their BMI was 24.99 ± 4.17, medical history of stroke, diabetes, and hyperlipemia was 26.1%, 56.8%, and 58.3% respectively; smoking and drinking accounted for 23.9% and 13.4%, respectively, while exercise accounted for 0.4%.

**TABLE 1:** General clinical characteristics, smoking, drinking, and exercise behavior of hypertensive patients

Variables	Male (*n* = 407)	Female (*n* = 362)	Total (*n* = 769)
Age, year	64.60 ± 11.85	66.86 ± 11.42	65.66 ± 11.70
BMI, kg/m^2^	25.27 ± 4.32	24.67 ± 3.98	24.99 ± 4.17
History of stroke	107(26.3)	94(26.0)	201(26.1)
History of diabetes	220(54.1)	217(59.9)	437(56.8)
History of hyperlipemia	263(64.6)	185(51.1)	448(58.3)
Smoking behavior	156(38.3)	28(7.7)	184(23.9)
Drinking behavior	87(21.4)	16(4.4)	103(13.4)
Lack of exercise	405(99.5)	361(99.7)	766(99.6)

### 3.2 Univariate analysis of stroke occurrence in hypertensive patients during 1‐year follow‐up

A total of 769 hypertensive patients were followed up for 1 year, and the incidence rate of stroke occurrence was 14.69%. The results in Table 2 showed that during 1‐year follow‐up, the increased incidence rates of stroke in hypertensive patients were related to the history of stroke, high age, abnormal index of blood pressure, blood glucose, TC, TG, HDL, HCY, aggravated extent of carotid artery stenosis, smoking, and drinking behavior (*p* < .05). Keeping exercise behavior decreased the incidence rate of stroke (*p* < .05). While the incidence rate of stroke has no significant difference between gender, overweight/obesity, and LDL (*p* > .05).

**TABLE 2:** Univariate analysis of stroke occurrence in hypertensive patients during 1‐year follow‐up

	Stroke (*n*/%)			
Variables	Onset	No onset	χ^2^	*p*
Gender				
Male	67(15.5)	340(83.5)	2.155	.142
Female	46(12.7)	316(87.3)		
Age				
<65	36(10.8)	298(89.2)	7.223	.007
≥65	77(17.7)	358(82.3)		
History of stroke				
Yes	56(27.9)	145(72.1)	37.633	.000
No	57(10.0)	511(90.0)		
Overweight/obese				
Yes	59(15.4)	325(84.6)	0.275	.600
No	54(14.0)	331(86.0)		
Blood pressure				
Normal	29(10.4)	251(89.6)	6.608	.010
Abnormal	84(17.2)	405(82.8)		
Blood glucose				
Normal	61(12.3)	434(87.7)	6.231	.013
Abnormal	52(19.0)	222(81.0)		
TC				
Normal	103(14.0)	632(86.0)	6.147	.013
Abnormal	10(29.4)	24(70.6)		
TG				
Normal	81(13.2)	531(86.8)	5.091	.024
Abnormal	32(20.4)	125(79.6)		
HDL‐C				
Normal	51(12.3)	363(87.7)	4.037	.045
Abnormal	62(17.5)	293(82.5)		
LDL‐C				
Normal	105(14.2)	634(85.8)	2.645	.104
Abnormal	8(26.7)	22(73.3)		
HCY				
Normal	5(3.1)	154(96.9)	21.331	.000
Abnormal	108(17.7)	502(82.3)		
Rate of carotid artery stenosis (%)				
Mild (<30)	50(8.6)	532(91.4)	109.421	.000
Moderate (30 ∼)	51(29.3)	123(70.7)		
Serious (70 ∼)	12(92.3)	1(7.7)		
Smoking				
Yes	28(33.3)	56(66.7)	26.135	.000
No	85(12.4)	600(87.6)		
Drinking				
Yes	19(43.2)	25(56.8)	30.215	.000
No	94(13.0)	631(87.0)		
Exercise				
Yes	5(4.4)	109(95.6)	11.346	.001
No	108(16.5)	547(83.5)		

### 3.3 Multivariate Cox regression analysis of stroke occurrence in hypertensive patients during 1‐year follow‐up

According to univariate analysis, a total of 12 variables (age, history of stroke, blood pressure, blood glucose, TC, TG, HDL‐C, HCY, rate of carotid artery stenosis, smoking, drinking, and exercise) entered the multivariate Cox regression analysis (forward method) in accordance with the inclusion (α = 0.05) and exclusion (β = 0.10) criteria. Results in Table 3 showed that the history of stroke (RR = 2.382, 95% CI: 1.605–3.534), higher levels of blood glucose (RR = 1.632, 95% CI: 1.110–2.400), TG (RR = 1.581, 95% CI: 1.026–2.434), HCY (RR = 3.702, 95% CI: 1.500–9.140), aggravated extent of carotid artery stenosis (RR = 2.677, 95% CI: 1.811–3.959), and smoking (RR = 2.440, 95% CI: 1.569–3.794) were risk factors for stroke occurrence in hypertensive patients, while maintaining exercise (RR = 0.260, 95% CI: 0.105–0.646) was a protective factor of stroke occurrence in hypertensive patients during 1‐year follow‐up.

**TABLE 3:** Multivariate Cox regression analysis of stroke occurrence in hypertensive patients during 1‐year follow‐up

Variables	*B*	SE	Wald	*p*	RR	95% CI
History of stroke	0.868	0.201	18.575	.000	2.382	1.605–3.534
Higher level of blood glucose	0.490	1.197	6.198	.013	1.632	1.110–2.400
Higher level of TG	0.458	0.220	4.315	.038	1.581	1.026–2.434
Higher level of HCY	1.309	0.461	8.058	.005	3.702	1.500–9.140
Moderate to Serious carotid artery stenosis	0.985	0.200	24.359	.000	2.677	1.811–3.959
Smoking	0.892	0.225	15.685	.000	2.440	1.569–3.794
Exercise	−1.347	0.464	8.414	.004	0.260	0.105–0.646

### 3.4 Univariate analysis of stroke occurrence in hypertensive patients without history of stroke during 1‐year follow‐up

A total of 568 hypertensive patients without history of stroke were followed up for 1 year, and the incidence rate of stroke was 10.04%. The results in Table 4 showed that during 1‐year follow‐up, the increased incidence rate of stroke in hypertensive patients without history of stroke was related to abnormal index of blood glucose, TC, TG, LDL, HCY, aggravated extent of carotid artery stenosis, smoking, and drinking behavior (*p* < .05). Keeping exercise behavior decreased the incidence of stroke (*p <* .05). While the incidence rate of stroke has no significant difference between gender, age, overweight/obesity, blood pressure, and HDL (*p* > .05).

**TABLE 4:** Univariate analysis of stroke occurrence in hypertensive patients without history of stroke during 1‐year follow‐up

	Stroke (*n*/%)			
factor	Onset	No onset	χ^2^	*p*
Gender				
Male	36(12.0)	264(88.0)	2.179	.099
Female	21(7.8)	247(92.2)		
Age				
<65	24(8.6)	255(91.4)	1.247	.264
≥65	33(11.4)	256(88.6)		
Overweight/obese				
Yes	36(11.6)	272(88.4)	1.806	.179
No	21(8.2)	236(91.8)		
Blood pressure				
Normal	18(8.4)	197(91.6)	1.060	.303
Abnormal	39(11.0)	314(89.0)		
Blood glucose				
Normal	24(7.1)	316(92.9)	8.311	.004
Abnormal	33(14.5)	195(85.5)		
TC				
Normal	50(9.3)	489(90.7)	5.187	.023
Abnormal	7(24.1)	22(75.9)		
TG				
Normal	35(8.1)	399(91.9)	7.914	.005
Abnormal	22(16.4)	112(83.6)		
HDL‐C				
Normal	34(10.2)	300(89.8)	0.019	.891
Abnormal	23(9.8)	211(90.2)		
LDL‐C				
Normal	51(9.4)	492(90.6)	4.147	.042
Abnormal	6(24.0)	19(76.0)		
HCY				
Normal	2(1.6)	124(98.4)	12.800	.000
Abnormal	55(12.4)	387(87.6)		
Rate of carotid artery stenosis (%)				
Mild (<30)	32(7.0)	427(93.0)	–	.000*
Moderate (30 ∼)	23(21.7)	83(78.3)		
Serious (70 ∼)	2(66.7))	1(33.3)		
Smoking				
Yes	18(26.1)	51(73.9)	22.415	.000
No	39(7.8)	460(92.2)		
Drinking				
Yes	13(38.2)	21(61.8)	31.856	.000
No	44(8.2)	490(91.8)		
Exercise				
Yes	4(4.3)	88(95.7)	3.933	.047
No	53(11.1)	423(88.9)		

* Regards as the exact probability method of Fisher.

### 3.5 Multivariate Cox regression analysis of stroke occurrence in hypertensive patients without history of stroke during 1‐year follow‐up

According to univariate analysis, a total of 10 variables (blood pressure, blood glucose, TC, TG, LDL‐C, HCY, rate of carotid artery stenosis, smoking, drinking, and exercise) entered the multivariate Cox regression analysis (forward method) in accordance with the inclusion (α = 0.05) and exclusion (β = 0.10) criteria. Results in Table 5 showed that higher levels of blood glucose (RR = 2.027, 95% CI: 1.195–3.438), HCY (RR = 5.928, 95% CI: 1.438–24.440),aggravated extent of carotid artery stenosis (RR = 2.620,95% CI: 1.532–4.481), and drinking (RR = 3.867, 95% CI: 2.038–7.339) were risk factors for stroke occurrence in hypertensive patients without history of stroke, while maintaining exercise (RR = 0.325, 95% CI: 0.117–0.907) was a protective factor of stroke occurrence in hypertensive patients without history of stroke during 1‐year follow‐up.

**TABLE 5:** Multivariate Cox regression analysis of stroke occurrence in hypertensive patients without history of stroke during 1‐year follow‐up

Factors	*B*	SE	Wald	*p*	RR	95% CI
Higher level of blood glucose	0.707	0.270	6.867	.009	2.027	1.195–3.438
Higher level of HCY	1.780	0.723	6.063	.014	5.928	1.438–24.440
Moderate to serious carotid artery stenosis	0.963	0.274	12.387	.000	2.620	1.532–4.481
Drinking	1.353	0.327	17.122	.000	3.867	2.038–7.339
exercise	−1.123	0.523	4.609	.032	0.325	0.117–0.907

### 3.6 Univariate analysis of stroke recurrence in hypertensive patients with history of stroke during 1‐year follow‐up

A total of 201 hypertensive patients with history of stroke were followed up for 1 year, and the recurrence rate of stroke was 27.86%. The results in Table 6 showed that during 1‐year follow‐up, the increased recurrence rate of stroke in hypertensive patients with history of stroke was related to abnormal index of blood pressure, blood glucose, HCY, aggravated extent of carotid artery stenosis, smoking, and drinking behavior (*p* < .05). Keeping exercise behavior decreased the incidence of stroke (*p <* .05). While the incidence of stroke has no significant difference between gender, age, overweight/obesity, TC, TG, HDL, and LDL (*p* > .05).

**TABLE 6:** Univariate analysis of stroke recurrence in hypertensive patients with history of stroke during 1‐year follow‐up

	Stroke(*n*/%)			
Factors	Occurrence	No occurrence	χ^2^	*p*
Gender				
Male	31(29.0)	76(71.0)	0.141	.708
Female	25(26.6)	69(73.4)		
Age				
<65	12(21.8)	43(78.2)	1.376	.241
≥65	44(30.1)	102(69.9)		
Overweight/obese				
Yes	23(31.5)	50(68.5)	0.758	.384
No	33(25.8)	95(74.2)		
Blood pressure				
Normal	11(16.9)	54(83.1)	5.718	.017
Abnormal	45(33.1)	91(66.9)		
Blood glucose				
Normal	37(23.9)	118(76.1)	5.364	.021
Abnormal	19(41.3)	27(58.7)		
TC				
Normal	53(27.0)	143(73.0)	1.250	.263
Abnormal	3(60.0)	2(40.0)		
TG				
Normal	46(25.8)	132(74.2)	3.152	.076
Abnormal	10(43.5)	13(56.5)		
HDL‐C				
Normal	17(21.3)	63(78.8)	2.890	.089
Abnormal	39(32.2)	82(67.8)		
LDL‐C				
Normal	54(27.6)	142(72.4)	0.012	.914
Abnormal	2(40.0)	3(60.0)		
HCY				
Normal	3(9.1)	30(90.9)	6.921	.009
Abnormal	53(31.5)	115(68.5)		
Rate of carotid artery stenosis (%)				
Mild (<30)	18(14.6)	105(85.4)	42.598	.000
Moderate (30 ∼)	28(41.2)	40(58.8)		
Serious (70 ∼)	10(100.0)	0(0.0)		
Smoking				
Yes	10(66.7)	5(33.3)	12.145	.000
No	46(24.7)	140(75.3)		
Drinking				
Yes	6(60.0)	4(40.0)	5.408	.020
No	50(26.2)	141(73.8)		
Exercise				
Yes	1(4.5)	21(95.5)	6.682	.010
No	55(30.7)	124(69.3)		

### 3.7 Multivariate Cox regression analysis of stroke recurrence in hypertensive patients with history of stroke during 1‐year follow‐up

According to univariate analysis, a total of 10 variables (blood pressure, blood glucose, TG, HDL‐C, HCY, rate of carotid artery stenosis, smoking, drinking, and exercise) entered the multivariate Cox regression analysis (forward method) in accordance with the inclusion (α = 0.05) and exclusion (β = 0.10) criteria. Results in Table 7 showed that aggravated extent of carotid artery stenosis (RR = 3.158, 95% CI: 1.797–5.550) and smoking (RR = 2.271, 95% CI: 1.142–4.517) were risk factors for stroke recurrence in hypertensive patients with history of stroke during 1‐year follow‐up.

**TABLE 7:** Multivariate Cox regression analysis of stroke recurrence in hypertensive patients with history of stroke during 1‐year follow‐up

Factors	*B*	SE	Wald	*p*	RR	95% CI
Moderate to serious carotid artery stenosis	1.150	0.288	15.977	.000	3.158	1.797–5.550
Smoking	0.820	0.351	5.466	.019	2.271	1.142–4.517

## 4 DISCUSSION

A total of 769 hypertensive patients were enrolled in this study. The average age of hypertensive patients was 65.66 ± 11.70 years and the BMI index was 24.99 ± 4.17. The older people are susceptible to high blood pressure due to organ failure or dysfunction. This study found medical history of stroke, diabetes, and hyperlipemia was 26.1%, 56.8%, and 58.3% respectively; smoking and drinking accounted for 23.9% and 13.4% respectively, while exercise accounted for 0.4% in hypertensive people. Studies have shown that hypertension is a major risk factor for stroke, followed by dyslipidemia, diabetes, physical inactivity, smoking, and overweight/obesity (Guan et al., 2017), while epidemiological studies have demonstrated a positive relationship between heavy alcohol use and hypertension (Stranges et al., 2004).

In this study, 769 patients with hypertension were followed up for 1 year, and the incidence of stroke was 14.69%. Data from the national stroke screening survey show that the standardized prevalence rate of survival stroke patients in study population aged 60 and older was 4.94% in total (Xia et al., 2019). Because hypertension was the most prevalent risk factor for stroke, people with high blood pressure have a higher stroke rate than the general population.

Multivariate Cox regression analysis of influencing factors of stroke in hypertensive patients followed up for 1 year revealed the history of stroke, higher levels of blood glucose, TG abnormal, HCY abnormal, aggravated extent of carotid artery stenosis, and smoking were risk factors, while maintaining exercise was a protective factor of stroke occurrence in hypertensive patients. Studies have shown that patients who survived their initial stroke had a significantly increased risk of further strokes compared to the general population (Mohan et al., 2011), people with diabetes have a largely increased risk of stroke compared with people without diabetes (Kvitkina et al., 2020), the incidence of acute ischemic stroke was significantly increased when there were low levels of TG and TG/HDL‐C (Deng et al., 2019), patients with hyperhomocysteinemia (HHcy) have a higher risk of developing ischemic stroke (Lu et al., 2019), carotid stenosis is a known independent risk factor for perioperative stroke (Klarin et al., 2020), smoking increases the risk of stroke in the general population, there is evidence of a dose‐response relationship between the number of cigarettes and the risk of stroke (Matsuo et al., 2020), and increases in physical activity levels may improve function and health status for stroke survivors (Aguiar et al., 2017).

In this study, hypertensive patients were divided into those with no history of stroke and those with history of stroke, and multivariate Cox regression analysis of influencing factors of stroke was conducted respectively. The 568 hypertensive patients without history of stroke were followed up for 1 year, and the incidence of stroke was 10.04%, while the rate of recurrent stroke in 201 patients with history of stroke was 27.86%. Another study from China shows that the rate of recurrent stroke after the first‐ever stroke was 5.7% (Han et al., 2020), and a meta‐analysis suggested that the cumulative risk of recurrence 1 year after initial stroke was 11.1% (Mohan et al., 2011); all of them were lower than the incidence of stroke in this study, probably because the subjects in this study were people with hypertension.

The study also found that higher levels of blood glucose, HCY, aggravated extent of carotid artery stenosis, and drinking were risk factors, and maintaining exercise was a protective factor for stroke occurrence; however, aggravated extent of carotid artery stenosis and smoking were risk factors for stroke recurrence for hypertensive patients. These risk factors were partly confirmed by other investigators. For instance, diabetes, smoking, dyslipidemia, and alcohol intake were recognized as important risk factors for the occurrence and recurrence of ischemic stroke in America and dyslipidemia and smoking were among the risk factors in Arab countries (Benamer & Grosset, 2009). Hypertensive patients without history of stroke had more risk factors than those with history of stroke, possibly because people with history of stroke have improved their bad behaviors, such as eating habits, drinking, and exercise.

From what has been discussed above, for people with high blood pressure, it is necessary to exercise properly, control body weight, avoid obesity, quit smoking, reduce alcohol consumption, and reasonably control blood pressure, blood sugar, and blood lipid.

Although this study is a prospective study, there are still some deficiencies. First, the main disadvantage of cohort study design is the existence of bias and confounding factors, which may affect the reliability of the study results. The patients with hypertension included in this study are from one hospital, which may have bias in admission rate. In addition, although this study has taken some measures to control potential confounding factors, we cannot completely rule out the interference of other unpredictable factors affecting the prognosis of patients. Second, this study only collected the physiological and biochemical indexes of patients at admission, and lacked the collection information during follow‐up.

### PEER REVIEW

The peer review history for this article is available https://publons.com/publon/10.1002/brb3.2770.

## CONFLICT OF INTEREST

The authors have no potential conflicts of interest to disclose.

## Data Availability

The data sets generated and analyzed during the current study are available from the corresponding author on reasonable request.
